# Polypharmacy-associated potential contraindications of drug prescriptions in patients with primary angle closure disease in a real-world setting

**DOI:** 10.1186/s40780-021-00196-w

**Published:** 2021-05-03

**Authors:** Fumiaki Tanaka, Naoki Shibatani, Kazumi Fujita, Hiroaki Ikesue, Satoru Yoshimizu, Nobuyuki Muroi, Yasuo Kurimoto, Tohru Hashida

**Affiliations:** 1 Department of Pharmacy, Kobe City Eye Hospital, 2-1-8 Minatojima-Minamimachi, Chuo-ku, Kobe, 650-0047 Japan; 2 Present Address: Department of Pharmacy, Kobe City Medical Center General Hospital, 2-1-1 Minatojima-Minamimachi, Chuo-ku, Kobe, 650-0047 Japan; 3 Department of Ophthalmology, Kobe City Eye Hospital, Kobe, Japan

**Keywords:** Primary angle closure disease, Angle closure, Glaucoma, Medications contraindicated for PACD, Polypharmacy

## Abstract

**Background:**

Primary angle closure disease (PACD) is a type of glaucoma in which the intraocular pressure (IOP) is increased because of the blockage of the anterior chamber angle. Medications contraindicated for patients with PACD, such as anticholinergics, cause mydriasis, and can elevate IOP. However, anticholinergics are currently contraindicated only for primary angle closure glaucoma (PACG) in Japanese package inserts. In this study, we investigated the prescription status of medications contraindicated for PACD, such as anticholinergics, in patients with PACD scheduled for eye surgeries.

**Methods:**

Forty-three Japanese patients diagnosed with PACD at Kobe City Eye Hospital, Japan, and scheduled hospitalization for eye surgeries between December 2017 and July 2018, were included. Data, including sex, age, diagnosis, IOP, anterior chamber depth, and patients’ regular medications prior to hospitalization, were collected for each patient from the electronic medical records.

**Results:**

The number of patients with chronic primary angle closure (CPAC) and acute primary angle closure (APAC) was 35 (81.4%) and 8 (18.6%), respectively. Among all the 43 patients with PACD, 8 (18.6%) received 15 medications that are potentially contraindicated for PACD by non-ophthalmologist. According to medication categories, benzodiazepine hypnotics were the most commonly prescribed. Among the 8 patients with APAC, 2 (25.0%) had routinely received medications contraindicated for PACD. The median number of all kinds of prescriptions on the day of hospitalization was significantly higher for patients who received medications contraindicated for PACD than for those who did not receive them (*p* = 0.010).

**Conclusions:**

About 20% of patients with PACD received medications potentially contraindicated for PACD, such as anticholinergics. Attention should be paid to patients prescribed multiple drugs for adverse events, such as increase in intraocular pressure.

## Background

Primary angle closure disease (PACD) is a type of glaucoma in which the intraocular pressure (IOP) is increased because of the blockage of the anterior chamber angle, where the aqueous outflow channel is located. It encompasses all classifications of primary angle closure suspect (PACS), primary angle closure (PAC), and primary angle closure glaucoma (PACG) [1]. PACG is a major cause of blindness in East Asia. In the Tajimi Study, a population-based epidemiological survey performed in Japan, the prevalence of PACD was reported to be 1.3% in residents aged 40 years or older [2]. In addition, the prevalence of PACG in the Kumejima Study was reported to be 2.2% in residents aged 40 years or older [3].

Patients with PACD are generally elderly, and therefore, polypharmacy is a particular concern for adverse events in these patients [4]. Medications contraindicated for patients with PACD, such as anticholinergics, cause mydriasis. Increased relative pupillary block induces the posterior part of the iris to bulge forward and plateau iris causes peripheral iris crowding with pupillary dilation. These mechanisms occlude the trabecular meshwork [5, 6]. Therefore, angle closure can elevate IOP if the patient does not undergo ophthalmic procedures, such as laser iridotomy, peripheral iridectomy, or lens extraction [7]. APAC is an ophthalmic emergency that can result in blindness if its recognition and treatment are delayed. Typical symptoms are ocular eye pain, headache, blurred vision, nausea, and vomiting [8, 9]. Anticholinergics can elevate IOP in patients with PACG [10–13]. Although anticholinergics are currently contraindicated only for PACG in Japanese package inserts, considering the mechanism of PACD, they should also be interpreted as contraindicated for patients with PAC and PACS [9, 14].

Although ophthalmologists carefully consider the condition and safety of patients when prescribing medications contraindicated for PACD, these medications are often prescribed by non-ophthalmologists. However, no study has been conducted to address this issue in patients with PACD. The current status of prescription of these medications in patients with PACD, including PAC and PACS, in a real-world setting has not been reported. Thus, we investigated the medications prescribed for patients with PACD who were admitted to Kobe City Eye Hospital, Japan.

## Methods

### Patients and setting

This retrospective study was conducted at Kobe City Eye Hospital, Japan. The study population consisted of 45 consecutive Japanese patients who were diagnosed with PACD at our hospital and were scheduled hospitalization for eye surgeries, including lens extraction and peripheral iridectomy, between December 2017 and July 2018. Patients with PACD were defined as those diagnosed with PACS, PAC, PACG, or APAC by ophthalmologists at our hospital. Patients with PACD in both the eyes were classified as having a more severe disease. For example, if a patient had PACS in one eye and PAC in the other, the case was classified as having PAC. PACS, PAC, and PACG with chronic clinical course were classified as chronic primary angle closure (CPAC). Among patients with CPAC, those who had previously been treated with ophthalmic procedures, such as laser iridotomy (*n* = 2), were excluded from the study.

### Data collection

The following data were retrospectively collected from the electronic medical records of Kobe City Eye Hospital for each patient: sex, age, diagnosis, IOP, anterior chamber depth (ACD), and patients’ regular medications prior to admission at our hospital. The IOP was measured by Goldmann applanation tonometry, and the ACD was measured by anterior segment optical coherence tomography (CASIA2; Tomey). The pharmacists confirmed the patients’ medication histories on the day of hospitalization by identifying each medication brought by the patient, interviewing the patients or their family members, reviewing their medication history booklets, and asking the community pharmacies which dispensed patients’ regular medications, as needed. We defined medications contraindicated for PACD referring to Japanese package inserts for each drug. The use of six or more medications by a patient was also defined as polypharmacy [4].

### Statistical analyses

Continuous data on age and number of prescribed medications are shown as median values and interquartile range (IQR), whereas continuous data such as IOP and ACD are shown as the mean ± standard deviation. Categorical data are shown as numbers (percentages). The number of prescriptions on the day of hospitalization of patients who received contraindicated drugs for PACD was compared to those who did not receive them using the Mann–Whitney *U* test. We used JMP version 13.0.0 (SAS Institute Inc., Cary NC, USA) for all statistical analyses and two-tailed *p*-values < 0.05 were considered statistically significant.

### Ethics

The protocol of this study was approved by the Ethics Committee of the Kobe City Medical Center General Hospital; the Committee waived the need for patients’ consent (approval no. ezn190101). This study was performed in accordance with the Declaration of Helsinki.

## Results

### Patient characteristics

Patient characteristics are summarized in Table 1. Among the 43 study subjects, the median age of the study population was 74 (IQR: 68–79) years, and 23.3% of the patients were male. The median number of medications for each patient on the day of hospitalization was 5 (IQR: 3–9). Twenty out of forty-three (46.5%) patients received more than six medications (polypharmacy). The number of patients with CPAC and APAC was 35 (81.4%) and 8 (18.6%), respectively. Among the patients with CPAC, 19, 13, and 3 were diagnosed with PACS, PAC, and PACG, respectively. Forty-one patients underwent surgery for lens extraction and two patients underwent peripheral iridectomy on the day of hospitalization or the day after hospitalization.

**Table 1:** Baseline characteristics

Characteristics	
Patients, n	43
Age, years	74 (68–79)
Male, n (%)	10 (23.3%)
Number of prescribed medicationson the day of hospitalization	5 (3–9)
Type of PACD	
Acute primary angle closure, n (%)	8 (18.6%)
IOP, mmHg*^1^	61.1 ± 11.2
ACD, mm	1.32 ± 0.21
Chronic primary angle closure, n (%)	35 (81.4%)
IOP, mmHg*^2^	18.0 ± 4.9
ACD, mm	1.87 ± 0.23
PACS, n (%)	19 (44.2%)
PAC, n (%)	13 (30.2%)
PACG, n (%)	3 (7.0%)

Continuous data on age and number of prescribed medications are shown as median values and interquartile range, whereas continuous data of IOP and ACD are shown as the mean ± standard deviation

*Abbreviations*: *PACD* primary angle closure disease, *PACS* primary angle closure suspect, *PAC* primary angle closure, *PACG* primary angle closure glaucoma, *IOP* intraocular pressure, *ACD* anterior chamber depth

*^1^: One patient was excluded because IOP was normal at hospitalization after using IOP-lowering medication at other hospital

*^2^: One patient was excluded because measurement of ACD was used non-contact tonometer

### Contraindicated medications for PACD

We investigated the regular medications taken by the patients on the day of their hospitalization. Eight (18.6%) out of 43 patients received medications contraindicated for PACD (Table 2). Among the eight patients with APAC, two (25.0%) patients routinely received medications contraindicated for PACD. The median number of all kinds of prescriptions on the day of hospitalization was significantly higher for patients who received medications contraindicated for PACD than for those who did not receive them (11 vs. 4 medications, *p* = 0.010).

**Table 2:** Number of patients who were taking medications contraindicated for primary angle closure disease

Type of PACD	Number of patients	Number of medications
Acute primary angle closure (*n* = 8)	2/8 (25.0%)	6
Chronic primary angle closure		
PACS (*n* = 19)	3/19 (15.8%)	3
PAC (*n* = 13)	1/13 (7.7%)	3
PACG (*n* = 3)	2/3 (66.7%)	3
TOTAL	8/43 (18.6%)	15

*Abbreviations*: *PACD* primary angle closure disease, *PACS* primary angle closure suspect, *PAC* primary angle closure, *PACG* primary angle closure glaucoma

The details of medications contraindicated for PACD are shown in Table 3. A total of 15 medications were prescribed for eight patients by non-ophthalmologists. According to the medication categories, hypnotics (*n* = 8) were the most prescribed, followed by medications for overactive bladder (OAB; *n* = 2), anxiolytics (*n* = 2), antiepileptics (*n* = 1), medication for Parkinson’s disease (*n* = 1), and multi-ingredient cold medication (*n* = 1).

**Table 3:** Medications contraindicated for patients with primary angle closure disease

Cases	Type of PACD	Patients’ regular medications	Alternative agents	Notes
1	APAC	Zolpidem	Ramelteon (melatonin receptor agonist) and/or suvorexant (orexin receptor antagonist)	None.
		Multi-ingredient cold medication (salicylamide, acetaminophen, caffeine and promethazine)	Acetaminophen	The necessity for antihistamines should be considered carefully. In view of lower kidney function (Ccr = 37.7 mL/min), careful consideration is also needed for prescribing NSAIDs.
2	APAC	Cloxazolam	NA	Because the patient took antidepressants, mirtazapine and sertraline, consultation with psychiatrist was considered for better control of the depressive disorder.
		Zolpidem and lormetazepam	Ramelteon (melatonin receptor agonist) and/or suvorexant (orexin receptor antagonist)	None
		Fesoterodine	Vibegron (adrenergic β_3_ agonist)	None
3	PACS	Eszopiclone	Ramelteon (melatonin receptor agonist) and/or suvorexant (orexin receptor antagonist)	None
4	PACS	Eszopiclone	Ramelteon (melatonin receptor agonist)	Because the patient took digoxin orally, replacement with suvorexant was not suitable (drug–drug interaction).
5	PACS	Eszopiclone	Ramelteon (melatonin receptor agonist) and/or suvorexant (orexin receptor antagonist)	None
6	PAC	Levodopa and carbidopa	NA	Changes to other drugs were difficult because of older age (77 years), and combination of the drug with dopamine agonist.
		Clonazepam	NA	Consultation with neurologist was recommended for better control of neurologic disease.
		Clotiazepam	NA	
7	PACG	Imidafenacin	Vibegron (adrenergic β_3_ agonist)	Because the patient took naftopidil, consultation with urologists was considered for better control of prostatic hyperplasia.
		Zolpidem	Ramelteon (melatonin receptor agonist) and/or suvorexant (orexin receptor antagonist)	None
8	PACG	Brotizolam	Ramelteon (melatonin receptor agonist) and/or suvorexant (orexin receptor antagonist)	None

*Abbreviations*: *PACD* primary angle closure disease, *APAC* acute primary angle closure, *Ccr* creatinine clearance, *NSAID* non-steroidal anti-inflammatory drug, *NA* not applicable, *PACS* primary angle closure suspect, *PAC* primary angle closure, *PACG* primary angle closure glaucoma

## Discussion

This is the first study to investigate the prescription status of medications, including anticholinergic drugs, for patients with PACD in the real-world setting. Medications contraindicated for PACD, such as anticholinergics for patients with angle closure, can cause mydriasis and increase the risk of elevated IOP [6–8]. Among all the 43 patients with PACD included in this study, 8 (18.6%) actually received 15 medications potentially contraindicated for PACD. Importantly, 25.0% (2/8) of the patients with APAC also received medications contraindicated for PACD.

Polypharmacy is associated with adverse drug events or increased mortality among the elderly [4, 15, 16]. In this study, the median number of all kinds of prescriptions on the day of hospitalization was significantly higher for patients who received medications contraindicated for PACD than for those who did not receive them. Medications contraindicated for PACD were not prescribed at our hospital but were prescribed previously by non-ophthalmologists. The safety concerns for medications, such as anticholinergics, are well recognized by ophthalmologists. Our results indicate that these safety concerns should be publicized widely among health care providers, including physicians and pharmacists, who provide medical care in various areas. We believe pharmacists can play an important role in maximizing patient safety with regard to the prescription of medications.

In this study, benzodiazepine hypnotics were found to be the most commonly prescribed drug category, followed by medications for OAB and anxiolytics. The continued medications contraindicated for PACD were no longer a significant problem for these patients, after resolving surgery for angle closure. Based on patients’ comorbidity, concomitant drugs, renal and hepatic function, clinical evidences, and pharmacological aspects, we considered alternative drugs in each case (Table 3). We believe that information about alternative drugs that do not increase IOP in patients with PACD should be provided to ophthalmologists. Among the hypnotic drugs, ramelteon, a melatonin receptor agonist, or suvorexant, an orexin receptor antagonist, were considered alternative drugs. In addition, benzodiazepines have been known to increase the risk of delirium, especially in older patients [17, 18]. Thus, switching benzodiazepine hypnotics to ramelteon or suvorexant in patients with PACD is recommended from the safety perspective. Only in case 2, who took digoxin orally, replacement with ramelteon was a better option to prevent an increase in serum digoxin concentration via the inhibition of intestinal P-glycoprotein due to the co-administration of digoxin and suvorexant [19]. Recently, the efficacy and safety of adrenergic β_3_ agonists, such as mirabegron and vibegron, in patients with OAB have been established [20, 21]. Mirabegron does not enhance pupillary block, theoretically, because it has no anticholinergic effect. However, a potential risk of increasing IOP is pointed out in the package insert of mirabegron because some cases of increased IOP have been reported abroad [22]. Therefore, switching anticholinergics for the treatment of OAB to vibegron in patients with PACD seems to be the most reasonable option.

There is a limitation to this study. This was a single-center, retrospective study involving a small number of patients. Further studies are warranted to evaluate the use of medications contraindicated for PACD by patients with PACD.

## Conclusions

This study demonstrates that about 20% of patients with PACD received medications potentially contraindicated for PACD, such as anticholinergics. Since drugs with anticholinergic effects, such as benzodiazepine hypnotics, are often prescribed for the elderly, attention should be paid to polypharmacy with regard to adverse events, such as an increase in IOP. Our findings may add a new perspective in the pharmacotherapy of patients with PACD.

## Data Availability

All data generated or analyzed during this study are included in this published article.

## Abbreviations

PACD: Primary angle closure disease
IOP: Intraocular pressure
CPAC: Chronic primary angle closure
APAC: Acute primary angle closure
PACS: Primary angle closure suspect
PAC: Primary angle closure
PACG: Primary angle closure glaucoma
IQR: Interquartile range
OAB: Overactive bladder
NA: Not applicable
Ccr: Creatinine clearance
NSAID: Non-steroidal anti-inflammatory drug
